# Medizinischer Bevölkerungsschutz in Deutschland – eine griechische Tragödie?

**DOI:** 10.1007/s10049-022-01100-1

**Published:** 2022-12-01

**Authors:** Matthias Bollinger

**Affiliations:** 1 Klinik für Anästhesiologie, Intensiv‑, Notfall- und Schmerzmedizin, Schwarzwald-Baar Klinikum, Klinikstr. 11, 78052 Villingen-Schwenningen, Deutschland; 2 Fakultät für Gesundheit – Lehrstuhl Anästhesiologie I, Universität Witten/Herdecke, Witten, Deutschland

**Keywords:** Bevölkerungsschutz/Reform, Massenanfall von Verletzten, Terrorismus, Katastrophenschutz, Pandemien, Civil protection/reform, Mass casualty incidents, Terrorism, Disaster management, Pandemics

## Abstract

Im Sinne des Bevölkerungsschutzes ist nichts dagegen einzuwenden, Ereignisse wie die Coronavirus-disease-2019-Pandemie selbstkritisch zu reflektieren und daraus Lehren zu ziehen. In der Vergangenheit hat dies aber häufig nicht dazu geführt, zukünftige Situationen und deren Konsequenzen zu antizipieren und sich besser vorzubereiten. Zum Teil mutet dies wie eine griechische Tragödie an: sehenden Auges in die Katastrophe – nur das dies nichts Schicksalhaftes an sich hat. Wichtige Szenarien, auf die der Bevölkerungsschutz in Deutschland nach Einschätzung des Autors unzureichend vorbereitet ist, sind neben Pandemien auch Anschläge und Unfälle mit CBRN-Gefahrstoffen (*CBRN* chemisch, biologisch, radiologisch, nuklear), Massenanfälle von Verletzten im Terrorfall mit Kliniken oder Rettungsdienst als Anschlagsziel sowie länger andauernde Strom- oder Trinkwasserausfälle und Einschränkungen in der Informations- und Kommunikationsstruktur nach Cyberattacken. Es ist dringend erforderlich, vorhandene Probleme zu benennen und funktionierende Konzepte für Krankenhäuser, Rettungsdienste und Schnelleinsatzgruppen einzufordern. Verantwortliche Behörden wiederum müssen Konzepte erarbeiten, finanzieren und umsetzen.

## Abstract (en)

In terms of civil protection, there is nothing against self-critical reflection of events, such as the coronavirus disease 2019 pandemic, and learning lessons from them. In the past, however, this has often not led to anticipating future situations, their consequences, and better preparation. To some extent, this seems like a Greek tragedy: conscious disregard—except that this must not be fate. In addition to pandemics, important scenarios for which civil protection in Germany is inadequately prepared, according to the authorʼs assessment, include attacks and accidents involving CBRN (chemical, biological, radiological, nuclear) hazardous substances, mass casualties in the event of a terror attack with hospitals or rescue services as the target of the attacks, as well as prolonged power or drinking water failures, and disruptions in information and communication structures following cyberattacks. There is an urgent need to identify existing problems and demand functional concepts for hospitals, rescue services, and rapid response teams. Responsible authorities, in turn, must develop, finance, and implement concepts.

Im Zusammenhang mit der Coronavirus-disease-2019(COVID-19)-Pandemie oder auch mit der Flutkatastrophe im Ahrtal wird gegenwärtig gerne der Begriff „lessons learned“ verwendet. Natürlich ist nichts dagegen zu sagen, wenn diese Ereignisse selbstkritisch reflektiert werden und man versucht, daraus Lehren zu ziehen. Tatsächlich aber sind diese oder ähnliche Schadensereignisse alle schon da gewesen, was jedoch bisher in vielen Fällen nicht dazu geführt hat, zukünftige Situationen und deren Konsequenzen zu antizipieren und sich besser bzw. ausreichend vorzubereiten.

Beispielhaft sei hier nur die kontinuierliche Beschäftigung des Bevölkerungsschutzes mit dem Szenario einer Pandemie seit spätestens 2003 genannt (Severe-acute-respiratory-syndrome-coronavirus-1[SARS-CoV-1]-Pandemie 2002/2003, Vogelgrippe 2004, Schweinegrippe 2009/2010, „Middle East respiratory syndrome coronavirus“ [MERS-CoV] 2012). Bereits 2005 wurde zum ersten Mal ein nationaler Pandemieplan des Robert Koch-Instituts (RKI) veröffentlicht [1]. Zwei Jahre später erfolgte eine länderübergreifende Übung des Bundesamts für Bevölkerungsschutz und Katastrophenhilfe (BBK; länder- und ressortübergreifende Krisenmanagementübung 2007 [LÜKEX 07]) mit dem Szenario einer Pandemie, in der wesentliche Problemfelder erkannt wurden [1]. Letztlich hat dies jedoch offensichtlich nicht dazu geführt, dass wir auf eine Pandemie ausreichend vorbereitet gewesen wären. Insbesondere zu Beginn der COVID-19-Pandemie war relativ schnell klar, dass es zum Teil an den einfachsten Dingen (Schutzkleidung, Desinfektionsmittel) fehlt. Die Severe-acute-respiratory-syndrome-coronavirus-2(SARS-CoV-2)-Pandemie sei hier jedoch nur als aktuellstes Beispiel genannt.

Zum Teil mutet dies für mich wie die Handlung einer griechischen Tragödie an: sehenden Auges in die Katastrophe – mit dem Unterschied, dass dies nichts Schicksalhaftes an sich hat. Vieles hätte man antizipieren können oder wusste es bereits aus den vorangegangenen Übungen und den Einschätzungen von Experten.

Weitere Szenarien, auf die wir meiner Einschätzung nach nur unzureichend vorbereitet sind:

- Anschläge und Unfälle mit CBRN-Gefahrstoffen (*CBRN* chemisch, biologisch, radiologisch, nuklear; beispielsweise der Saringasanschlag in Tokyo 1995 oder der vereitelte Rizinanschlag in Köln 2018): Nach Empfehlung einer Bund-Länder-Arbeitsgruppe wurden ab 2006 Dekon-V-Einheiten für die Dekontamination von Verletzten eingeführt [2]. Diese Einheiten sind jedoch in der Regel nur an wenigen Standorten stationiert. Nach einer gegebenenfalls längeren Anfahrt zum Einsatzort benötigen die Einheiten mindestens 30 min, um mit der Dekontamination der ersten Patienten beginnen zu können. Eine Einheit kann pro Stunde planmäßig 10 liegende und 40 gehfähige Patienten dekontaminieren. Als alternative Strategie hat sich in einigen Kommunen eine Zusammenarbeit mit Gefahrstoffzügen von Feuerwehren entwickelt. Wobei auch diese Zusammenarbeit nicht regelhaft beübt wird und somit die Erfahrung im Umgang mit kontaminierten Patienten fehlt. Die meisten Kliniken verfügen weder über ortsfeste noch über aufstellbare Möglichkeiten einer Dekontamination [3, 4] und sind auf derartige Ereignisse mutmaßlich nur unzureichend vorbereitet, obwohl insbesondere die Erfahrungen aus Tokyo gezeigt haben, dass sich eine große Anzahl Kontaminierter unmittelbar nach dem Ereignis selbstständig im nächsten Krankenhaus vorstellt. Zudem wird die Feuerwehr bei einem entsprechenden Schadensfall regelhaft keine freien Ressourcen haben, um eine Dekontamination am Krankenhaus durchzuführen.
- Massenanfall von Verletzten und Erkrankten im Terrorfall (Terror-MANV) mit Kliniken oder Rettungsdienst als Anschlagsziel: Zwischen 1981 und 2013 gab es international rund 100 Anschläge auf Kliniken [5, 6], beim Anschlag auf das Bataclan 2015 in Paris wurde das Hôpital Necker vorsorglich von der Polizei abgeriegelt. Es existiert zwar eine „Handlungsempfehlung zur Eigensicherung für Einsatzkräfte der Katastrophenschutz- und Hilfsorganisationen bei einem Einsatz nach einem Anschlag“ [7], diese ist aber eher allgemein gehalten und lässt aus Sicht des Autors viele Fragen unbeantwortet. Verantwortlich sind formal die Länder bzw. Landkreise und kreisfreien Städte. In Ermangelung klarer Konzepte übergeordneter Stellen haben sie zumeist selbst Konzepte für derartige Lagen entwickelt. Reale Einsätze und Übungen – wo durchgeführt – zeigen jedoch immer wieder Schwachstellen auf. Unabhängig davon werden in der nichtpolizeilichen Gefahrenabwehr derartige Lagen aber auch zu selten bis gar nicht geübt, mit den sich daraus ergebenden Konsequenzen. Für die Krankenhäuser existiert kein Schutzkonzept, da polizeiliche Kräfte bei einer derartigen Lage eine andere Prioritätensetzung verfolgen.
- Länger andauernde Strom- oder Trinkwasserausfälle sowie Einschränkungen in der Informations- und Kommunikationsstruktur nach Cyberattacken sind nur ein Teil anderer Bedrohungen für die Infrastruktur von Rettungsdiensten und Krankenhäusern. Für Krankenhäuser besteht die Pflicht, einen Krankenhaus-Alarm- und -Einsatzplan zu erstellen. Aktualität, Inhalte oder Funktionieren dieser Pläne werden in den meisten Bundesländern jedoch nicht von den Behörden kontrolliert und die meisten Bundesländer stellen auch keine Gelder zur Aufstellung, Umsetzung oder regelmäßigen Beübung solcher Pläne zur Verfügung. Eine Kostendeckung über die Krankenkassen ist nach aktueller Gesetzeslage vermutlich nicht zulässig.

Für all diese Szenarien fehlen aus Sicht des Autors schlüssige und funktionierende Gesamtkonzepte. Wo solche existieren, werden sie nicht ausreichend umgesetzt bzw. finanziert. Daneben existieren weitere bekannte, aber letztlich ungelöste Problemfelder, die die Bewältigung von Schadenslagen immer wieder kompliziert gestalten, wie etwa Unterschiede in der Aufbauorganisation der Führung von polizeilicher und nichtpolizeilicher Gefahrenabwehr und die Kommunikation zwischen diesen, Mangel an einheitlichen Strukturen in den verschiedenen Bundesländern und Verantwortungsdiffusion zwischen Behörden der zum Teil verschiedenen Verwaltungsebenen (Ämter der Landkreise, Innenministerien der Länder). Ein weiteres Problem ist das Fehlen von Einsatzkräften aufgrund eines zunehmenden Rückgangs längerfristigen ehrenamtlichen Engagements einerseits und fehlender Strukturen für die Einbindung von Spontanhelfern andererseits. Der fehlende Dank, der den Ärztinnen und Ärzten, dem Pflege- und Rettungsdienstpersonal und anderen Helfern in der COVID-19-Pandemie meiner Einschätzung nach entgegenzubringen versäumt wurde, mag dies nicht verbessern.

Die letzte umfangreiche Umstrukturierung im medizinischen Bevölkerungsschutz war der Aufbau von Schnelleinsatzgruppen zu Beginn der 1990er-Jahre, der weitgehend aus den Hilfsorganisationen initiiert war. Seitdem hat es nur kleinere Veränderungen in der Einsatztaktik gegeben. Nun wurde 2021 eine Neuausrichtung des BBK in die Wege geleitet. Vielleicht ein Zeichen, dass ein politischer Wille zur Verbesserung der bestehenden Strukturen aktuell vorhanden ist. Kritisch anzumerken ist jedoch, dass das Kernproblem des BBK nicht die mangelnde Expertise ist, sondern der unverbindliche Charakter erarbeiteter Empfehlungen gegenüber den einzelnen Bundesländern, die für den Katastrophenschutz zuständig sind. Dies spiegelt sich dann in eher unkonkreten Anforderungen in Landesrettungsdienst- bzw. Landeskrankenhausgesetzen wider.

Man kann aus Problemen lernen, man kann diese aber auch antizipieren. Es ist daher dringend erforderlich, dass die notfallmedizinischen Fachgesellschaften und Hilfsorganisationen die vorhandenen Probleme benennen und funktionierende Konzepte für Krankenhäuser, Rettungsdienste und Schnelleinsatzgruppen mit Nachdruck einfordern. Auf der anderen Seite ist es erforderlich, dass die verantwortlichen Behörden Konzepte erarbeiten, diese finanzieren und umsetzen, auf ihr Funktionieren überprüfen und schließlich fortlaufend beüben lassen.
